# Modeling and Analyzing the Mutual Inductance of Rogowski Coils of Arbitrary Skeleton

**DOI:** 10.3390/s19153397

**Published:** 2019-08-02

**Authors:** Xiaoyu Liu, Hui Huang, Chaoqun Jiao

**Affiliations:** School of Electrical Engineering, Beijing Jiaotong University, Beijing 100044, China

**Keywords:** Rogowski coil, shape of the skeleton, current measurement, shape of the primary conductors

## Abstract

There are Rogowski coils of various shapes in the on-site measurement, and it is difficult to calculate the electrical quantities of Rogowski coils of curved skeleton and circular cross-section by simulation software. This paper proposes a theoretical derivation to calculate the mutual inductance between the conductors of any shape and Rogowski coils with skeletons of any shape. Based on the derivation, the influence of four skeleton shapes of Rogowski coils and four shapes of the primary conductors on the mutual inductance of Rogowski coils are studied by the comparison between the ideal cases and some non-ideal ones. The gap and gap compensation of the openable Rogowski coils are also considered. Experiments verify the numerical results according to the derivation. It is shown that to reduce the errors of the measurement the circular skeleton deformation should be avoided, the coil’s skeleton should be with curved angle, the primary conductor should be as straight as possible and should go through the center of the skeletons vertically. Furthermore, for the Rogowski coils of the rectangular skeleton, we propose a new skeleton structure to reduce the deviation influence of the primary conductors.

## 1. Introduction

Rogowski coils, which are also called current measurement coils or differential current sensors, are designed to measure different types of alternating and transient current (from tens to several thousands of amperes) [1]. Traditional electromagnetic current sensors cannot accurately measure high Ampere currents because of the saturation problem of the magnetic core [2], while the Rogowski coil is different: Its skeleton is made of non-ferromagnetic materials, and its turns evenly wound on the skeleton [3]. The Rogowski coil has been widely used in multiple industries [4,5] due to its characteristics—simplicity, low cost, light weight, linearity, dynamic bandwidth, non-invasive and immunity for core saturation [6,7,8,9,10].

The operating principle of the Rogowski coil is based on Ampere and Faraday laws [11]. When a Rogowski coil is around an alternating current-carrying conductor, the coil generates an inductive voltage [1]. Mutual inductance is a crucial parameter between the inductive voltage and the primary conductors [12,13,14,15]. Therefore, to study the influence quantities on mutual inductance is important to ensure the accuracy of Rogowski coil [16,17].

Antonio Cataliotti et al. present the results of an experimental study dealing with the characterization and error compensation of a commercial Rogowski coil. The influence of the position of the primary conductor on the errors of experimental results is analyzed [18]. Mirko Marracci et al. present the results of an analytical and experimental study of the critical parameters affecting the mutual inductance between the straight primary conductors and the Rogowski coil with the rectangular cross-section of the circular skeleton, while the study focuses on the case when the primary conductors are straight and located in the hole of the coil, the shape of skeleton is circular and the cross-section of the circular skeleton is rectangular [19]. The effects of the geometrical parameters on high frequency performance of Rogowski coil are studied in [20], and the mutual inductance is also given of the Rogowski coils with the rectangular, circular and oval cross-sections and the primary conductors of the infinite length in the paper. In [21], the mutual inductance between the discrete Rogowski coils and the conductors with the circular and rectangular cross-sections is calculated by the magnetic vector potential method. The influence of the section parameters, the tilt angles, and the eccentricity of the conductors on the errors of the mutual inductance is analyzed, when their conductors are straight and of the infinite length and their shapes of the cross-sections are rectangular. The influence of the adjacent external conductors on the mutual inductance of the Rogowski coil is studied in [2], and when the primary conductors are straight and of the infinite length. Mengyuan Xu et al. analyze the influence of the position of the primary conductors on the measurement errors of a circular array of the magnetic sensors for current measurement, and the straight and infinitely long primary conductors are located inside of the circular array of the magnetic sensors [22]. A special experiment is carried out to prove that the mutual inductance of PCB Rogowski coil is immune to the variation of the center of the plasma current in [4], and the formula of mutual inductance applies to the cases with the infinite-length primary conductors and the uniform magnetic field on the skeleton section. Luka Ferkovi$\overset{´}{c}$ et al. present the influence of the primary conductor from the central position of the Rogowski coil, and their studies focus on the case with the infinite-length straight conductors and the Rogowski coil with the rectangular cross-sections [23,24]. Mario Chiampi et al. investigate the effects of some influence quantities on flexible Rogowski coil, and the numerical model is based on the magnetic vector potential for cases with the straight primary conductor and the circular skeleton of the Rogowski coil [25]. Tao et al. present the calculation and measurement of mutual inductance of the PCB Rogowski coil under several conditions, including the eccentricity from axis, external parallel and perpendicular of the primary conductors [26].

Unfortunately, it is difficult to get the mutual inductance of the Rogowski coil by 3D electromagnetic simulation software because of the large number of its turns and the huge difference in the sizes between its enameled wire (usually the diameter of the cross-section is less than 0.17 mm) and the primary conductors (usually the side of the cross-section is larger than 120 mm). Therefore, the theoretical derivation and experimental study for the mutual inductance between the conductor and the Rogowski coil are effective approaches to figure out the influence quantities.

**Figure 1:**
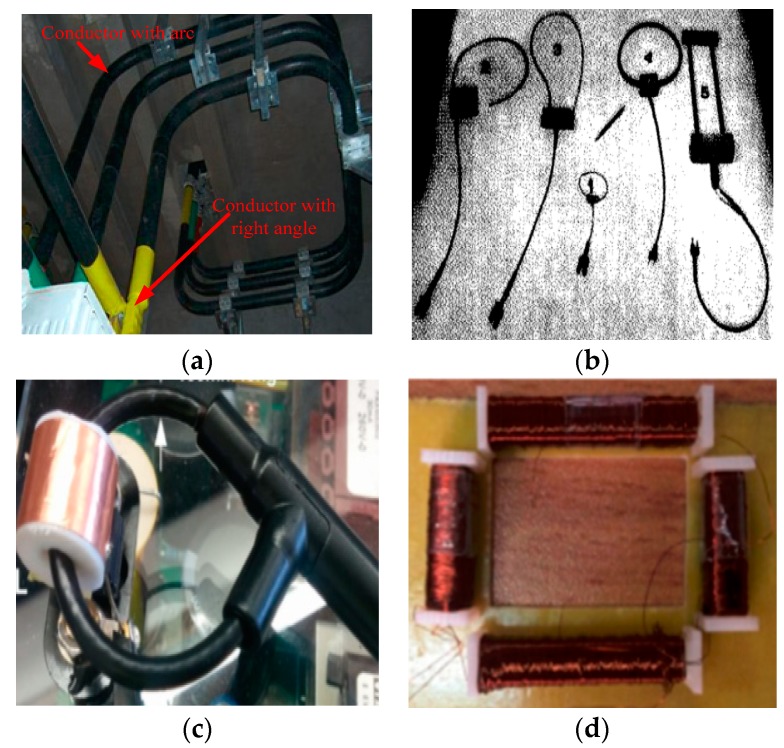
Conductors and Rogowski coils. (**a**) Conductors with some shapes; (**b**) five Rogowski coils of different structures; (**c**) non-circular Rogowski coil; (**d**) rectangular Rogowski coil.

Since there are various shapes of the conductor and Rogowski coil in the on-site measurement [27] (Figure 1 shows some cases), it is crucial to figure out the mutual inductance between the Rogowski coil with the skeleton of any shape and the primary conductor of different shapes in order to reduce the measurement errors. Here, we present a mathematical model to calculate the mutual inductance between the Rogowski coil with the skeleton of any shape and the primary conductor of any shape. Moreover, we compare the results of the experimental and numerical calculation, which are based on our theoretical derivation for 3 kinds of Rogowski coils and the conductors with several common shapes. Moreover, we present a new structure of rectangular skeletons with arc angle corner to reduce the errors. The numerical calculation results show that this structure can greatly decrease the errors (about 12.6%) of mutual inductance caused by the position and cross-section of the primary conductors.

## 2. Theoretical Derivation of Mutual Inductance

The basic Rogowski coil relation is [28]

(1) v = −Mdidt

where *v* and *i* are the instantaneous values of the induce voltage of the Rogowski coil and the primary current respectively, and *M* is the mutual inductance between the Rogowski coil and the primary conductor.

### 2.1. Rogowski Coils with Arbitrary Skeleton and Straight Conductors

To study the mutual inductance between the arbitrary primary conductor and the Rogowski coil of the arbitrary shape skeleton, we begin with what goes between the straight conductor and the Rogowski coil with the arbitrary shape skeleton.

**Figure 2:**
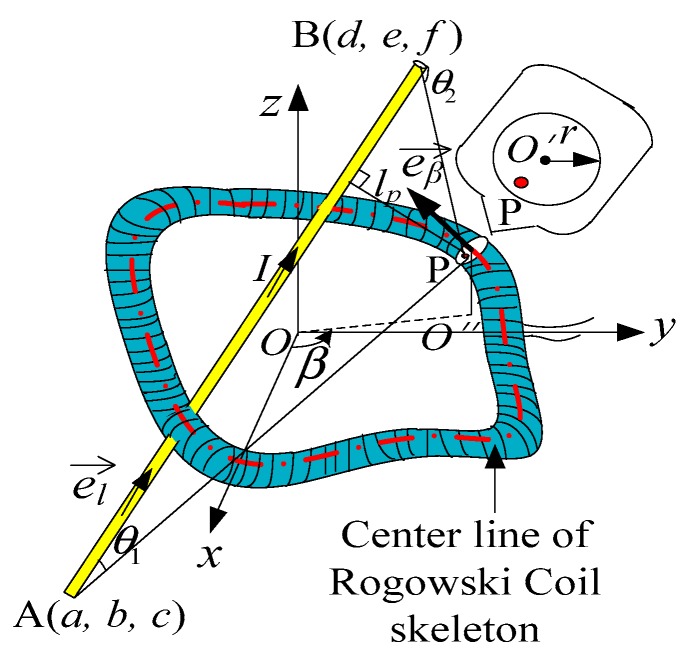
Structure diagram of a Rogowski coil of arbitrary shape and a finite-length straight conductor.

As shown in Figure 2, the cross-section of the skeleton is a circle with radius *r*. We pick the center of the cross-section of a turn as *O*’*. O*” is the projection of point *O*’ onto the *xOy* plane. The angle between *x*-axis and *O*’ *O*” is β, as shown in Figure 2. Then we pick a point P on the cross-section arbitrarily. A(a,b,c) and B(d,e,f) are the ends of the straight conductor. $l_{P}$ is the vertical distance from point P to the straight conductor. The angle between the conductor AB and line AP is *θ*_1_, while the angle between the conductor BA and line BP is *θ*_2_. *N* is the number of the turns of a Rogowski coil.

Here, we assume that the parameter equation of the center line of the distorted coil skeleton is:

(2) $$\{\begin{array}{c} x\;=\;\chi \left(\beta \right)\\ y\;=\;\varpi \left(\beta \right)\\ z\;=\;\psi \left(\beta \right) \end{array}\;(0<\beta \leq 2\pi )$$

The normal vector of the coil cross-section where point P is located is ${\vec{e}}_{\beta }\left(\chi^{\prime }\left(\beta \right),\varpi^{\prime }\left(\beta \right),\psi \prime \left(\beta \right)\right)$.

The coordinate equation of point P on the cross-section of the turn which corresponds to O′(χ(β),ϖ(β),ψ(β)) is

(3) $$\left\{ \begin{array}{l} x_{P}\;=\;\chi \left(\beta \right)+r\left(\frac{\varpi^{\prime }\left(\beta \right)\cos t}{\sqrt{{\left(\chi^{\prime }\left(\beta \right)\right)}^{2}+{\left(\varpi^{\prime }\left(\beta \right)\right)}^{2}}}+\frac{\chi^{\prime }\left(\beta \right)\psi^{\prime }\left(\beta \right)sint}{\sqrt{{\left(\chi^{\prime }\left(\beta \right)\psi^{\prime }\left(\beta \right)\right)}^{2}+{\left(\varpi^{\prime }\left(\beta \right)\psi^{\prime }\left(\beta \right)\right)}^{2}+{\left({\left(\chi^{\prime }\left(\beta \right)\right)}^{2}+{\left(\varpi^{\prime }\left(\beta \right)\right)}^{2}\right)}^{2}}}\right)\\ y_{P}\;=\;\varpi \left(\beta \right)+r\left(\frac{-\chi^{\prime }\left(\beta \right)\cos t}{\sqrt{{\left(\chi^{\prime }\left(\beta \right)\right)}^{2}+{\left(\varpi^{\prime }\left(\beta \right)\right)}^{2}}}+\frac{\omega^{\prime }\left(\beta \right)\psi^{\prime }\left(\beta \right)sint}{\sqrt{{\left(\chi^{\prime }\left(\beta \right)\psi^{\prime }\left(\beta \right)\right)}^{2}+{\left(\varpi^{\prime }\left(\beta \right)\psi^{\prime }\left(\beta \right)\right)}^{2}+{\left({\left(\chi^{\prime }\left(\beta \right)\right)}^{2}+{\left(\varpi^{\prime }\left(\beta \right)\right)}^{2}\right)}^{2}}}\right)\;\left(\begin{array}{c} 0<t\leq 2\pi ,\\ \;0<r\leq r_{0} \end{array}\right)\\ z_{P}\;=\;\psi \left(\beta \right)-\frac{r\left({\left(\chi^{\prime }\left(\beta \right)\right)}^{2}+{\left(\varpi^{\prime }\left(\beta \right)\right)}^{2}\right)sint}{\sqrt{{\left(\chi^{\prime }\left(\beta \right)\psi^{\prime }\left(\beta \right)\right)}^{2}+{\left(\varpi^{\prime }\left(\beta \right)\psi^{\prime }\left(\beta \right)\right)}^{2}+{\left({\left(\chi^{\prime }\left(\beta \right)\right)}^{2}+{\left(\varpi^{\prime }\left(\beta \right)\right)}^{2}\right)}^{2}}} \end{array}\right.$$

The magnetic density magnitude of point P generated by the current I flowing in the straight conductor AB is given by [29]:

(4) $$B_{P}=\;\mu_{0}I\left(\cos\theta_{1}-\cos\theta_{2}\right)/4\pi l_{P}$$

where $\mu_{0}$ is the permeability of air, which equals 4 × 10^−7^ H/m.

The normal vector of ${\vec{B}}_{P}$ is

(5) $${\vec{e}}_{B}\;=\;\left(\vec{e_{l}}\times \vec{PA}\right)/\left|\vec{e_{l}}\times \vec{PA}\right|$$

According to Equations (4) and (5), we can get the projection of the magnetic density onto the normal vector $\vec{e_{\beta }}$

(6) $$B_{P\beta }\;=\;B_{P}\cdot (({\vec{e}}_{\beta }\cdot {\vec{e}}_{B})/(\left|{\vec{e}}_{\beta }\right|\left|{\vec{e}}_{B}|)\right)$$

**Figure 3:**
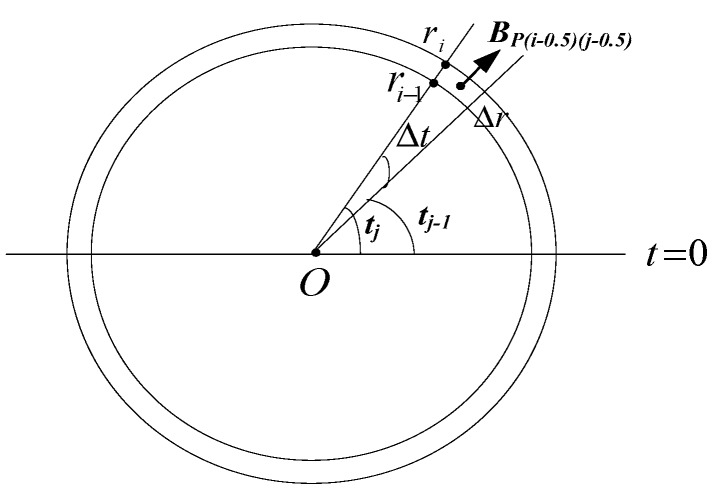
The magnetic field of one point on a circular cross-section of one turn.

In order to get the magnetic flux of the cross-section of the turn, which corresponds to angle β, we divide $r_{0}$ and 2π into equal parts $n_{1}$ and $n_{2}$, respectively. As shown in Figure 3, we get that $\Delta r\;=\;\frac{r_{0}}{n_{1}},\;\Delta t\;=\;\frac{2\pi }{n_{2}}$. $B_{P\beta \left(i-0.5\right)\left(j-0.5\right)}$ $\left(i\;=\;1,2,\ldots ,n_{1},\;j\;=\;1,2,\ldots ,n_{2}\right)$ means the magnetic flux of point P located in the section (r = (i−0.5)Δr, t = (j−0.5)Δt).

According to Equations (3) and (6), the magnetic flux of the cross-section of the *k*th turn corresponding to angle β is

(7) $$\Phi_{\beta k}\;=\;\overset{n_{1}}{\underset{i\;=\;1}{\sum }}\overset{n_{2}}{\underset{j\;=\;1}{\sum }}B_{P\beta \left(i-0.5\right)\left(j-0.5\right)}r\Delta r\Delta t$$

According to Equation (7), the magnetic flux of all the turns of the Rogowski coil is

(8) $$\Phi \;=\;\overset{N}{\underset{k\;=\;1}{\sum }}\Phi_{\beta k}$$

where *βk* is the angel *β* corresponding to the *k*th turn of the Rogowski coil.

According to Equation (8), mutual inductance *M* of the Rogowski coil with the straight primary conductor is [21]

(9) M = ΦI

### 2.2. Rogowski Coils with Arbitrary Skeleton and Curved Conductors

**Figure 4:**
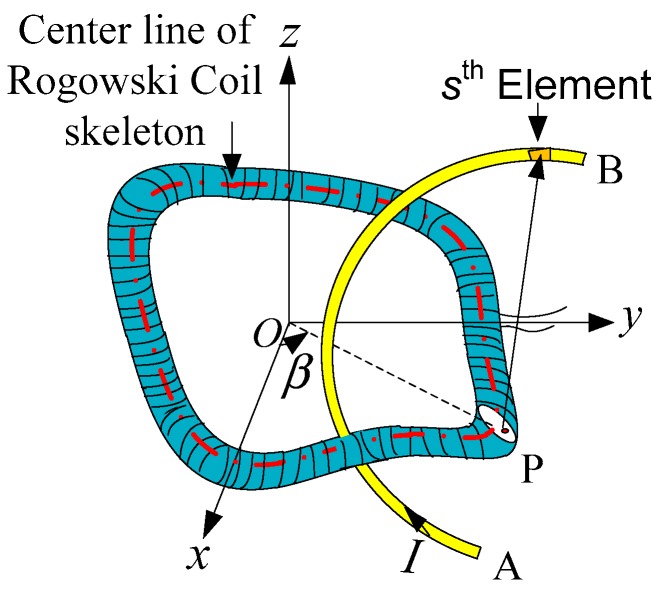
Structure diagram of a Rogowski coil and a finite-length curvilinear conductor.

To study the influence of the curvilinear conductor in the on-site measurement on mutual inductance of the Rogowski coil of arbitrary shape skeleton, we deduce an expression of mutual inductance between the Rogowski coil of arbitrary shape skeleton and the curvilinear conductor in this section, as shown in Figure 4.

Divide the conductor into infinitesimal elements. The magnetic field generated by the *s*th current element of the curve at point P is given by [30,31]

(10) $$B_{pm}\;=\;(\mu_{0}I\vec{dl_{s}}\times \vec{l_{s}^{\prime }})/\left(4\pi {\left|l_{s}^{\prime }\right|}^{3}\right)$$

The magnetic flux on the turn corresponding to angle β generated by the *s*th current element is $\Phi_{\beta s}$, and we can get $\Phi_{\beta s}$ from Equations (8) and (10). The magnetic flux of the whole turn of the Rogowski coil generated by the *s*th current element is $\Phi_{s}$, and we can get $\Phi_{s}$ from Equation (8). The magnetic flux of the whole coil generated by the whole conductor is given by

(11) $$\Phi \;=\;\underset{s}{\sum }\Phi_{s}$$

Mutual inductance *M* of the Rogowski coil with the curvilinear primary conductor can be obtained according to Equation (9).

## 3. Experimental Platform

**Figure 5:**
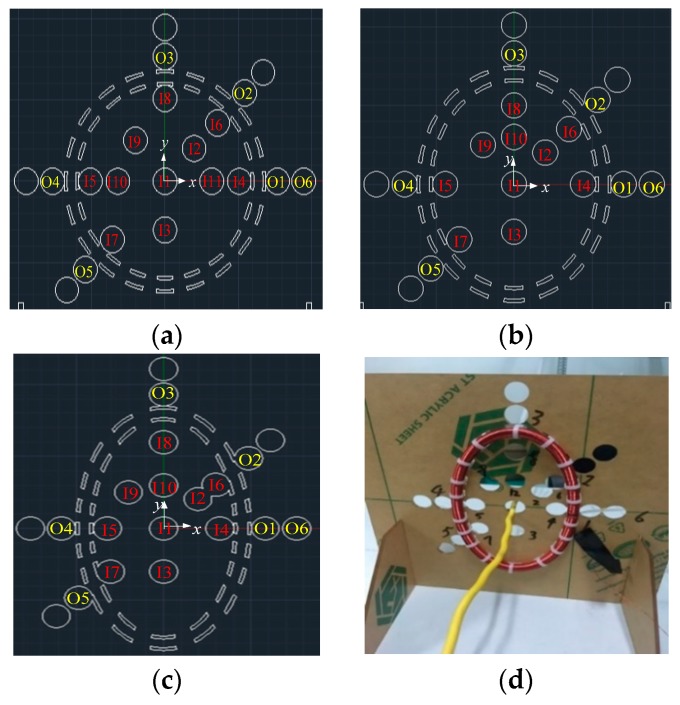
Fixed plates with test holes. (**a**) The fixed plate of Coil A; (**b**) the fixed plate of Coil B; (**c**) the fixed plate of Coil C; (**d**) the picture of the test plate.

**Figure 6:**
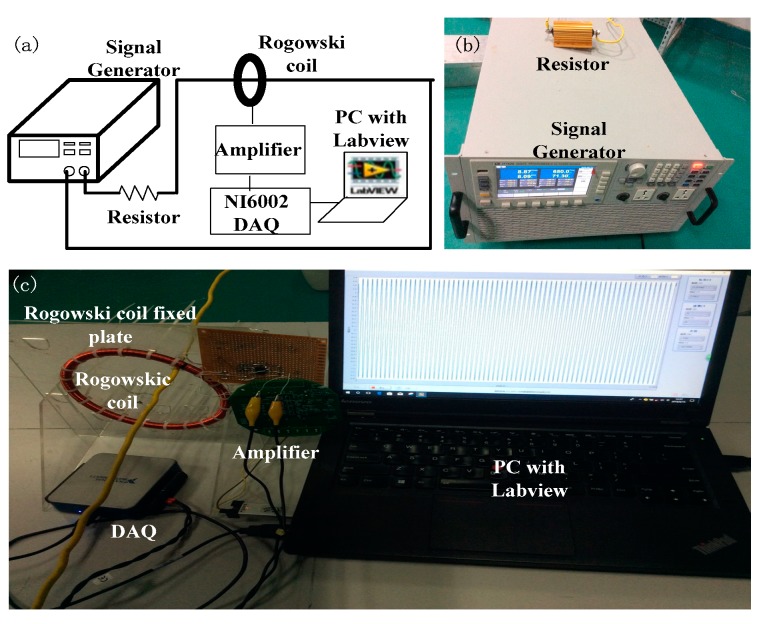
Experimental platform. (**a**) Schematic diagram; (**b**) power supply and the resistor; (**c**) experimental equipment in the laboratory environment.

To verify the derivation which we deduced above, we build the experimental platform, as shown in Figure 5 and Figure 6, to study the mutual inductance between the Rogowski coils of non-circular skeleton and the primary conductors of any shape, size and position. We wind three Rogowski coils by the electric winding machine to simulate the condition in which a flexible Rogowski coil deforms under external forces. Coil A is of circular skeleton and gap compensation, while both Coil B and Coil C are of oval skeleton and gap compensation [25], as shown in Figure 5. The three Rogowski coils have the same circumstance, cross-sections and turn numbers. The three Rogowski coils are fixed on three acrylic plates with the test holes to hold the coils in shape and place. The parameters of the three Rogowski coils are shown in Table 1: *N* is the number of the turns of the Rogowski coil, *r* is the radius of the cross-section of the coil skeleton, *L*_g_ is the length of the gap of the Rogowski coil, *N*_cg_ is the turn number of the gap compensation, and $\beta_{g}$ is the angle between the line from the middle point of the gap to the center of the skeleton O and *x*-axis. Table 2 shows the information of the test holes which are located in the primary conductors of the fixed plates, while holes label I1, I2, I3, …, I11 and O1, O2, O3, …, O6 represent the inner holes and the outside holes respectively on the fixed plate. In order to reduce the interference of the coils, the nearest charged body is 2.5 m away from the coils in the experiments.

As shown in Figure 6, we use ITECH IT7626 AC programmable power supply to generate the sinusoidal voltage signal with frequency of 100Hz and amplitude of 0~10 V. The resistance value of the resistance is 1 Ω. The power supply, the resistance and the primary conductor with the insulation form the AC current loop. The amplifier OP07 is to amplify the induction voltage signal at both ends of the Rogowski coils and to transmit the amplified signal to the acquisition card NI 6002 DAQ. The amplification coefficient of the amplifier is 100. DAQ transmits signal to PC with LabVIEW software with filter function.

## 4. Results of Numerical Calculation and Experiments

To verify the mutual inductance formula we deduced above, we calculated the mutual inductance between the Rogowski coils of the circular, oval and rectangular skeleton and the primary conductors of several common shape by MATLAB code according to the derivation in Section 2. The results of the calculation of the Rogowski coils of the oval and circular skeletons were verified by the experiments.

The circumference of the center line of the Rogowski coils of the rectangular and oval skeleton we chose was the same as that of the Rogowski coil of the circular skeleton (Coil A). The number of turns and the radius of the cross-section of the Rogowski coil of the rectangular skeleton were equal to those of Coil A, respectively. Moreover, we assumed that the turns were uniformly distributed on the skeleton. We defined Coil D as the Rogowski coil of the rectangular skeleton, and the length of the quadrilateral center line was 50.1 mm.

*M*_0_, which has been studied in [32], presents the mutual inductance of the Rogowski coil of the circular skeleton in the ideal case (the primary conductor of infinite-length goes perpendicularly through the center of the Rogowski coil of the circular cross-section and circular skeleton) as a standard to normalize the results of the nonideal cases.

**Figure 7:**
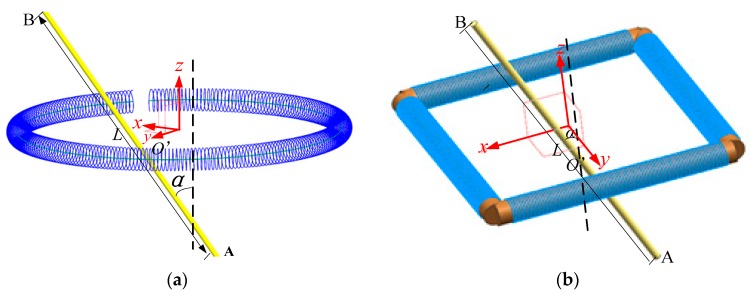
Conductor nonvertically through Rogowski coils (*xOy* plane) (λ = 0.5). (**a**) Rogowski coil of oval skeleton; (**b**) Rogowski coil of rectangular skeleton.

All calculation results by MATLAB codes are prefixed with “Cal.” and the experimental measurement results are prefixed with “Mea”. All center lines of the Rogowski coil skeleton in Figures 7, 10, 12, 15 and 18 are on the *xOy* coordinate plane.

### 4.1. Rogowski Coils of Noncircular Skeleton and Long Straight Primary Conductors

While the primary conductor is apt to tilt or deviate from the center line (*z*-axis as shown in Figure 7) of the Rogowski coil, the circular skeleton of the flexile Rogowski coils are easy to deform under the external force when the Rogowski coils are used. We studied the influence of the straight primary conductor tilted or deviated from the center of the skeleton on the mutual inductance of the Rogowski coils of the oval or rectangular skeletons. All the Rogowski coils have both gap and gap compensation in this section.

As shown in Figure 7, the intersection of the primary conductor AB and the *xOy* plane is $O^{\prime }$. Parameter λ is the ratio of A*O*’ and AB; λ = 0.5 means that the middle of the primary conductor is on the *xOy* plane; *L* is the length of the primary conductor, and *L* = 5000 mm, and *α* is the angle of the primary conductor tilted from the *z-*axis.

**Figure 8:**
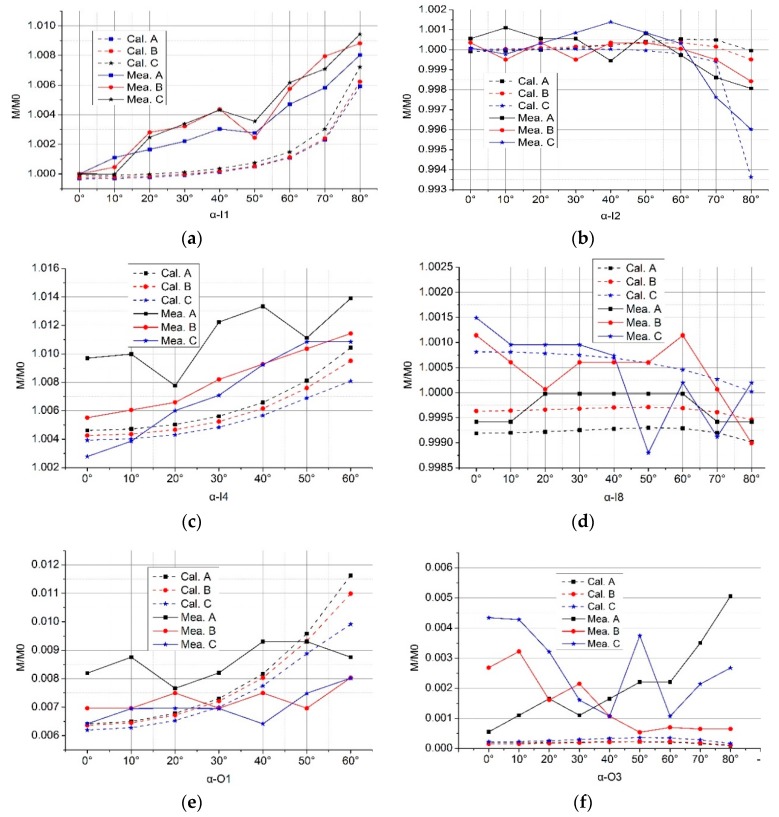
Normalized mutual inductance vs. tilted angle when the straight primary conductor does not go vertically through different positions of Coil A, Coil B, Coil C. (**a**) At inner hole I1; (**b**) at inner hole I2; (**c**) at inner hole I4; (**d**) at inner hole I8; (**e**) at outer hole O1; (**f**) at outer hole O3.

We show both the calculation and experimental results of several cases in Figure 8. As we can see, they are matched. Normalized mutual inductance of the Rogowski coil of the oval skeleton increases (up to 1.01) as the tilted angle *α* increases when the primary conductor goes through the inner holes I1 and I4. While in the inner holes I2 and I8, it decreases as the angle *α* increases. Under the same condition, the mutual inductance changes (up to 7%) when the skeleton of the Rogowski coil is deformed. Figure 8e,f shows the interference of the external conductor in the coils when the skeletons are deformed. Results show that serious deformation of coil skeleton should be avoided, and the primary conductor should avoid being close to the gap of the Rogowski coil.

**Figure 9:**
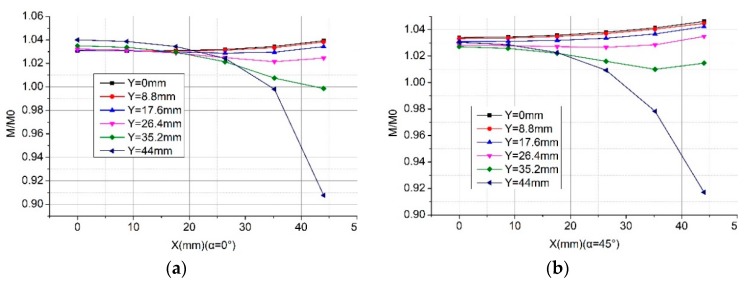
Calculated normalized mutual inductance vs. position when the primary conductor goes nonvertically through the Coil D. (**a**) α = 0°; (**b**) α = 45°.

For Coil D whose skeleton is rectangular, we show the calculation results in Figure 9. Moreover, we can see the mutual inductance of the Rogowski coil of the rectangular skeleton is larger (about 3.075%) than that of the coil of the circular skeleton (with the same skeleton circumference and number of turns). The closer the primary conductor to the corner of the rectangular skeleton, the greater the mutual inductance changes. The mutual inductance of the Rogowski coil of the rectangular skeleton fluctuates more than that of the oval or circular skeleton.

### 4.2. Rogowski Coils of Noncircular Skeleton and External Conductors Parallel to the Coil Plane(xOy)

**Figure 10:**
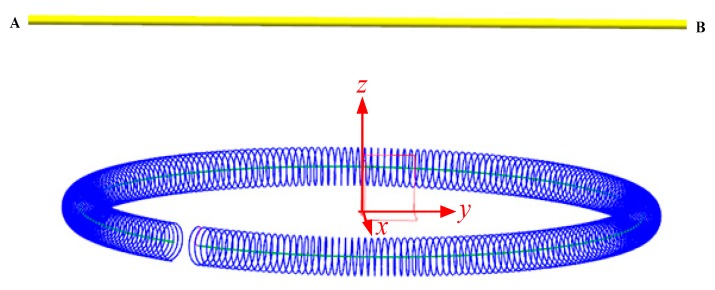
The external conductor parallel to the plane of the Rogowski coils of the oval skeleton.

In this section, we study the sensitivity of the Rogowski coils of different skeleton shape to the external conductor parallel to the coils. As shown in Figure 10, the external conductor is parallel to the *y*-axis and the distance *h* of the conductor to the coils is 8 mm. The length of the external conductor is 5000 mm.

**Figure 11:**
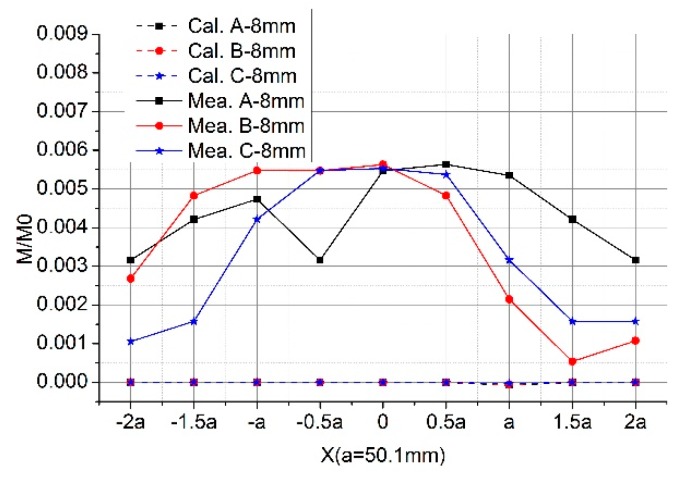
Normalized mutual inductance vs. position primary conductor of the when the conductors are parallel to the Rogowski coils plane.

According to the results shown in Figure 11, the parallel conductor has little effect on the mutual inductance even for the deformed skeleton case. Moreover, the parallel conductor should not be placed right above the plane of the Rogowski coils.

### 4.3. Rogowski Coils of Noncircular Skeleton and D-Shaped Conductors

**Figure 12:**
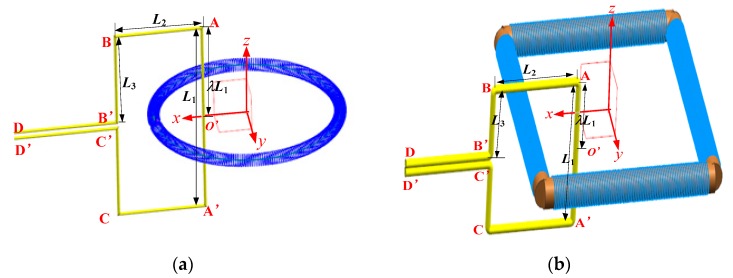
The D-shaped primary conductor vertically through Rogowski coils (λ = 0.5). (**a**) The Rogowski coil of the oval skeleton; (**b**) the Rogowski coil with rectangular skeleton.

In this section, we explore the influence of the primary conductor with the right angles on the mutual inductance of the Rogowski coils. As shown in Figure 12, the lengths of the conductor section AB, AA’, A’C and BC are all equal to 117.8 mm. The lengths of BB’, CC’ and AO’ are equal to 58.9 mm. The conductors DB’ and D’C’ are tightly tied together, and the magnitude of the currents in DB’ and C’D’ are equal while their directions are opposite. Therefore, the sum of the magnetic flux produced by DB’ and C’D’ is zero.

**Figure 13:**
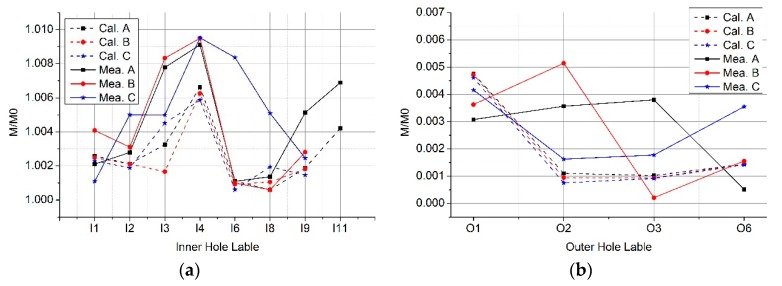
Normalized mutual inductance between the Rogowski coils and the D-shaped primary conductors. (**a**) The D-shaped conductor through the inner holds; (**b**) the D-shaped conductor through the outer side holds.

With the D-shaped primary conductor, we show both the calculation and experimental results for the Rogowski coils of the circular or oval skeleton in Figure 13. As shown in Figure 13a, the mutual inductance between the D-shaped primary conductor and the coils changes (up to 0.3%) when the skeleton of Rogowski coil is deformed. The mutual inductance of the D-shaped conductor is greater than that of the long straight primary conductor. When the primary conductor goes through the inner holes, the D-shaped conductor has greater effect on the mutual inductance than that of the long straight conductor, while the outside D-shaped conductor has less effect.

**Figure 14:**
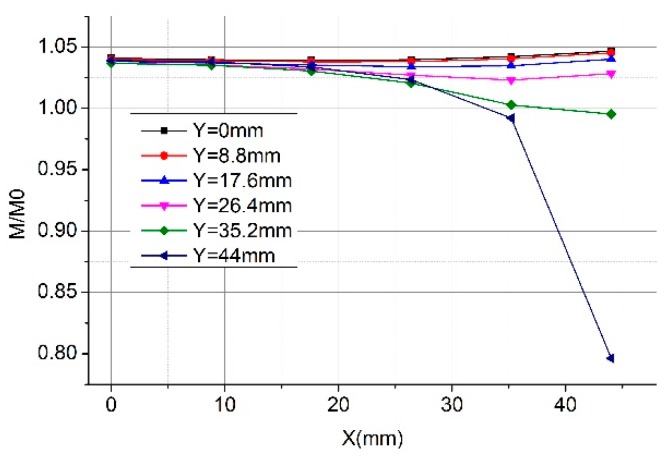
Calculation normalized mutual inductance between the Rogowski coil of rectangular skeleton and the D-shaped primary conductor.

Also with a D-shaped primary conductor, we show the calculation results for the Rogowski coil of the rectangular skeleton in Figure 14. The mutual inductance between the Rogowski coil of the rectangular skeleton and the D-shaped primary conductor is larger than that of the long straight conductor and Coil A. The influence of the D-shaped conductor on the mutual inductance of Coil D is larger than that of the long straight primary conductor when the position of the primary conductor changes. The closer the D-shaped conductor to the corner of the rectangular skeleton, the greater the influence on the mutual inductance (up to 25%).

### 4.4. Rogowski Coils of Noncircular Skeleton and O-Shaped Conductors

**Figure 15:**
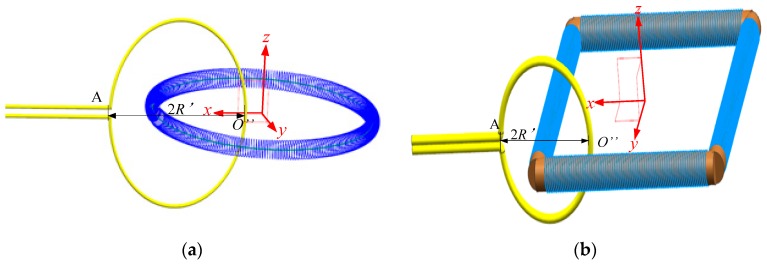
Curved conductor going vertically through the coils. (**a**) The Rogowski coil of the oval skeleton; (**b**) the Rogowski coil of the rectangular skeleton.

In this section, we study the mutual inductance between the curved primary conductor and the Rogowski coil with deformed skeleton. As shown in Figure 15, $2R^{\prime }\;=\;150\;\mathrm{mm}$ is the diameter of the O-shaped conductor. The conductor plane is parallel to the *xOz* plane and is symmetric about the *xOy*. $O^{\prime \prime }$ is the intersection of the conductor and the *xOy* plane.

**Figure 16:**
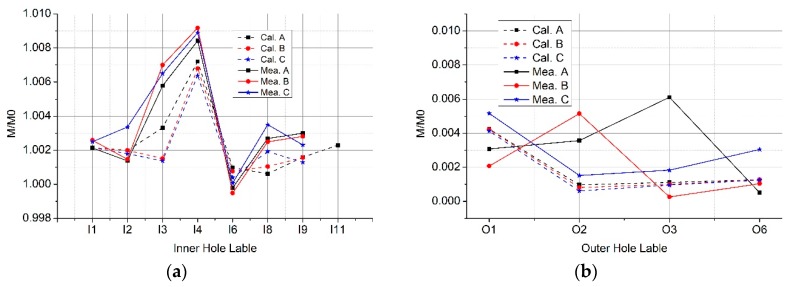
Normalized mutual inductance between the Rogowski coils and the O-shaped primary conductors. (**a**) The O-shaped conductor going through the inner holes; (**b**) the O-shaped conductor going through the outer holes.

Both the calculation and experimental results are shown in Figure 16. From Figure 16a, we can see that the mutual inductance of the O-shaped conductor is greater (0.2%) than that of the long straight primary conductor. The error of the mutual inductance between the O-shaped primary conductor and the Rogowski coil is greater (up to 0.3%) when the skeleton of the Rogowski coil is deformed. The primary conductor should not be close to the gap of the Rogowski coil. The outside O-shaped conductor has less effect on the mutual inductance of the Rogowski coil than that of the outside long straight conductor. The skeleton of the flexible Rogowski coil should be kept from deforming.

**Figure 17:**
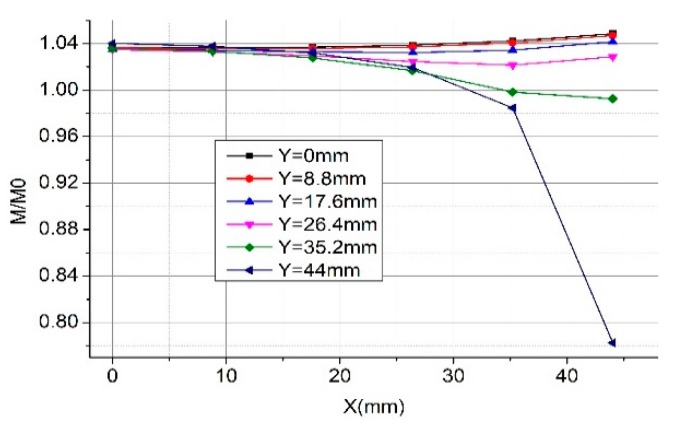
Calculation of the normalized mutual inductance between the Rogowski coil of the rectangular skeleton and the O-shaped primary conductors.

Figure 17 shows that the mutual inductance between the Rogowski coil of the rectangular skeleton and O-shaped primary conductor is larger than that of the long straight conductor. The influence of the O-shaped conductor on the mutual inductance is larger than that of the long straight primary conductor when the position of the primary conductor changes. The closer the O-shaped conductor to the corner of the rectangular skeleton, the greater the influence on the mutual inductance (up to 30%). The curved primary conductor should be avoided in this case.

### 4.5. Improved Rogowski Coils of Rectangular Skeleton

**Figure 18:**
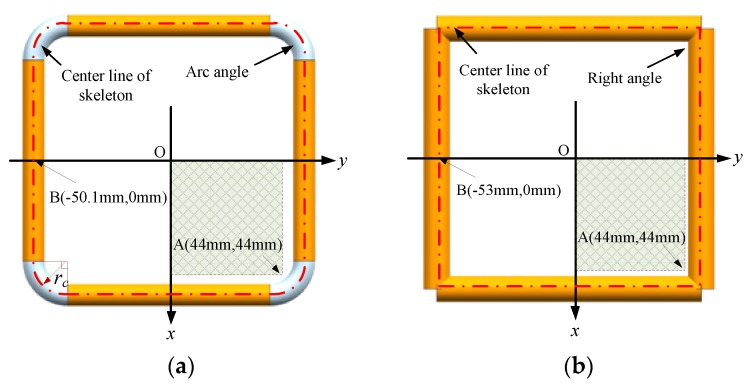
Skeletons with circular cross-section of the Rogowski coil. (**a**) Skeleton with arc angle; (**b**) skeleton with right angle.

It is shown in Figure 18 that the mutual inductance of the Rogowski coil of the rectangular skeleton of the right angle turns large when the primary conductor deviates from the center of the skeleton. Errors of the mutual inductance of the coils with the right-angle skeleton are also produced from the changes of the cross-section of the primary conductors [21].

**Figure 19:**
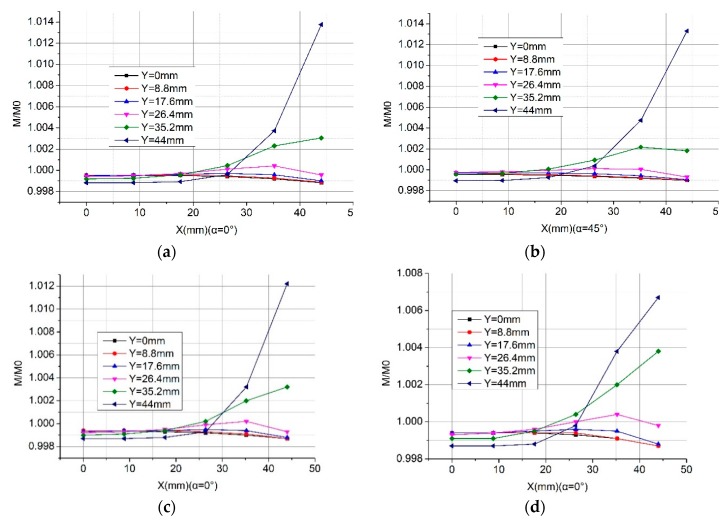
Calculation results of the normalized mutual inductance between the Rogowski coil of the rectangular skeleton and the long straight primary conductor. (**a**) Skeleton with an arc angle and primary conductor ($\alpha \;=\;0{}^{\circ};\;r_{c}\;=\;13.369\;\mathrm{mm}$); (**b**) skeleton with an arc angle and tilt angle of the primary conductor (α = 45°; $r_{c}\;=\;13.369\;\mathrm{mm}$); (**c**) skeleton with an arc angle and the primary conductor (α = 0°; $r_{c}=$ 15.9155 mm); (**d**) skeleton with an arc angle and the primary conductor (α = 0°; $r_{c}=$ 22.2817 mm).

Here, we propose a new structure of the Rogowski coil of the rectangular skeleton which can greatly reduce the deviation influence. As shown in Figure 18a, we changed the right angle of the rectangular skeleton into an arc angle, and the circumstance and turns number of the center line of the skeleton with an arc angle continues to be the same as that of those with a right angle. $r_{c}$ is the radius of the arc angles. To study the influence of curvature radius $r_{c}$ on mutual inductance, we set different radius $r_{c}$ as shown in Figure 19.

We show the calculation results of the new structure with different tilt angles of the primary conductor in Figure 19. Compared with the corresponding results of the right-angle structure in Figure 9, the new structure is stronger to keep the mutual inductance even when the position of the primary conductor changes. For example, at the corner position (point A: 44 mm, 44 mm), the mutual inductance of the right-angle structure changes 13% (shown in Figure 9) while that of the arc angle changes 1.4%. Furthermore, the new structure is not sensitive to the tilt angle of the primary conductor since Panels a and b of Figure 19 are alike. Figure 19a,c,d shows that the larger the curvature radius is, the less the displacement of the conductors affect the mutual inductance of the Rogowski coils of rectangular skeleton.

## 5. Conclusions

In this paper, we established a mathematic model to calculate the mutual inductance between the Rogowski coils of skeletons of arbitrary shape and the primary conductors of arbitrary shape at arbitrary position to verify the correctness of the derivation and the influence of the skeletons on the mutual inductance of the Rogowski coils. The mutual inductance between the Rogowski coils of different skeleton shapes and conductors of common shapes are calculated by MATLAB codes which we have written based on the mathematic models. Some calculated results were verified by the experiments. For the Rogowski coil of the rectangular skeleton, we propose a new structure to reduce the deviation influence.

According to the calculation and experimental results, there are some ways to reduce the error of mutual inductance: Deformation should be avoided in the skeletons of flexible Rogowski coils, the primary conductors should be located close to the center of the skeletons and the primary conductors should be as straight as possible. The rectangular skeletons with the arc angle of the Rogowski coils can also greatly reduce the change of the mutual inductance when the position and shape of the primary conductor change.

Although we have presented the test results of the common cases in this paper, the model we have established above is suitable for any shape and position. This allows engineers to design optimal Rogowski coils according to the shapes and sizes of the primary conductors without doing many experiments.

## Figures and Tables

**Table 1:** Parameters of the three test coils.

Coil Label	*N*	*r* (mm)	*a* (mm)	*b* (mm)	*L*_g_ (mm)	*N*_cg_	*β_g_*
Coil A	1604	4.3	63.8	63.8	2	10	0°
Coil B	1604	4.3	57.3	70	2	10	0°
Coil C	1604	4.3	45.2	80	2	10	0°

**Table 2:** Location information of the holes of the fixed plates.

Coil Label	Coil A		Coil B		Coil C	
Position	*x* (mm)	*y* (mm)	*x* (mm)	*y* (mm)	*x* (mm)	*y* (mm)
I1	0	0	0	0	0	0
I2	20	20	20	20	20	20
I3	0	−30	0	−30	0	−30
I4	51	0	44	0	32	0
I5	−51	0	−44	0	−32	0
I6	36	36	35	35	30	30
I7	−36	−36	−35	−35	−30	−30
I8	0	51	0	51	0	60
I9	−20	25	−20	25	−20	25
I10	−32	0	0	30	0	30
I11	32	0	-	-	-	-
O1	77	0	70	0	58	0
O2	54	54	54	54	49	48
O3	0	77	0	83	0	93
O4	−77	0	−70	0	−58	0
O5	−54	−54	−54	−54	−49	−49
O6	95	0	88	0	76	0
